# Supplementary material for: The effects of coating culture dishes with collagen on fibroblast cell shape and swirling pattern formation
Source: J Biol Phys. 2020 Aug 29;46(4):351–69. doi: 10.1007/s10867-020-09556-3 (PMC7719137; doi:10.1007/s10867-020-09556-3)
Supplement: Supplementary file 1 — (DOCX 1528 kb) [file 10867_2020_9556_MOESM1_ESM.docx]

**SUPPLEMENTARY INFORMATION**

**The effects of coating culture dishes with collagen on fibroblast cell shape and swirling pattern formation**

Kei Hashimoto^1,2,3,†^, Kimiko Yamashita^1,2,4,5,†^, Kanako Enoyoshi^1,2,†^, Xavier Dahan^2^, Tatsu Takeuchi^6^, Hiroshi Kori^1,7,^*, and Mari Gotoh^3^

^1^Graduate School of Humanities and Sciences, Ochanomizu University, Ohtsuka, Bunkyo-ku, Tokyo, Japan

^2^Program for Leading Graduate Schools, Ochanomizu University, Ohtsuka, Bunkyo-ku, Tokyo, Japan

^3^Institute for Human Life Innovation, Ochanomizu University, Ohtsuka, Bunkyo-ku, Tokyo, Japan

^4^Department of Physics, National Tsing Hua University, Hsinchu, Taiwan

^5^Physics Division, National Center for Theoretical Sciences, Hsinchu, Taiwan

^6^Department of Physics, Virginia Tech, Blacksburg, VA 24061, USA

^7^Department of Complexity Science and Engineering, Graduate School of Frontier Sciences, The University of Toyo, Kashiwa, Japan

*Correspondence should be addressed to H. Kori (kori@k.u-tokyo.ac.jp)

**Supplementary Figure S1**

The concentration of coated collagen on the well surface. The ratio of coated collagen type-I concentration on the well after incubating with 0 (uncoated control, only the 1 mM HCl vehicle solution), 0.1, 1.0 and 10.0 μg/mL collagen type-I solutions. The concentrations are normalized to that of the uncoated well. Data represent the mean ± SEM for each of the four initial collagen concentrations.

**Supplementary Figure S2**

**
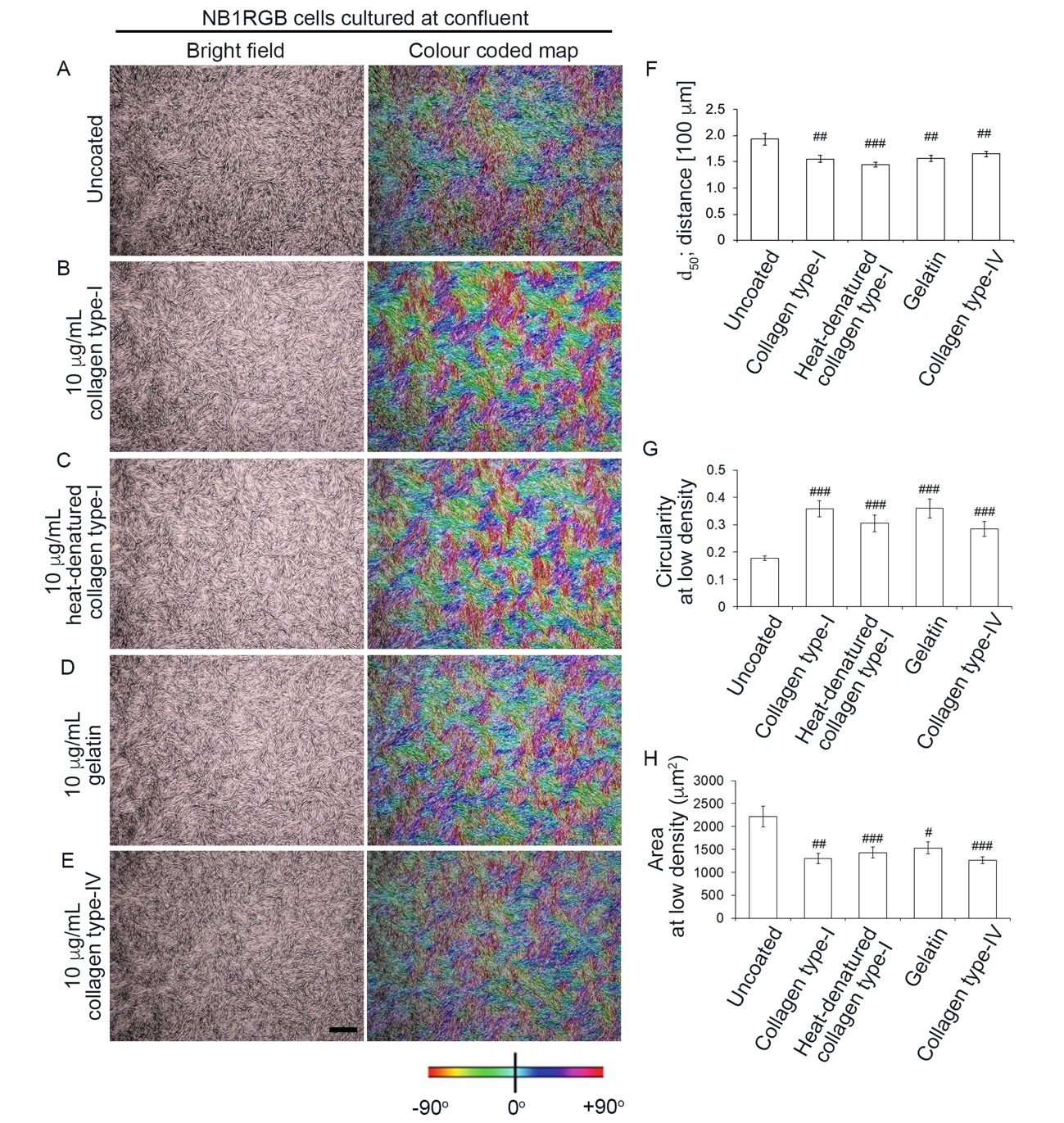
**

The effects of various types of collagen on the pattern formation of human-skin fibroblasts, NB1RGB, cultured for 72h on polystyrene dishes. (A–E) Bright-field images and colour coded maps of cultured NB1RGB cells at confluence on uncoated (control) (A), 10.0 μg/mL collagen type-I-coated (B), 10.0 μg/mL heat-denatured collagen type-I-coated (C), 10.0 μg/mL gelatin-coated (D), and 10.0 μg/mL collagen type-IV-coated (E) dishes. Scale bar: 1 mm. (F) $d_{50}$values at 72 h. #: *p* < 0.05, ##: *p* < 0.01, ###: *p* < 0.001, *t*-test vs. the $d_{50}$value of control. (G, H) Circularity and area of NB1RGB cultured at 24 h. #: *p* < 0.05, ##: *p* < 0.01, ###: *p* < 0.001, *t*-test vs. control value. Correlation data represent the mean ± SEM of 12 images from 4 dishes. Other data represent the mean ± SEM of 20 cells.

**Supplementary Figure S3**


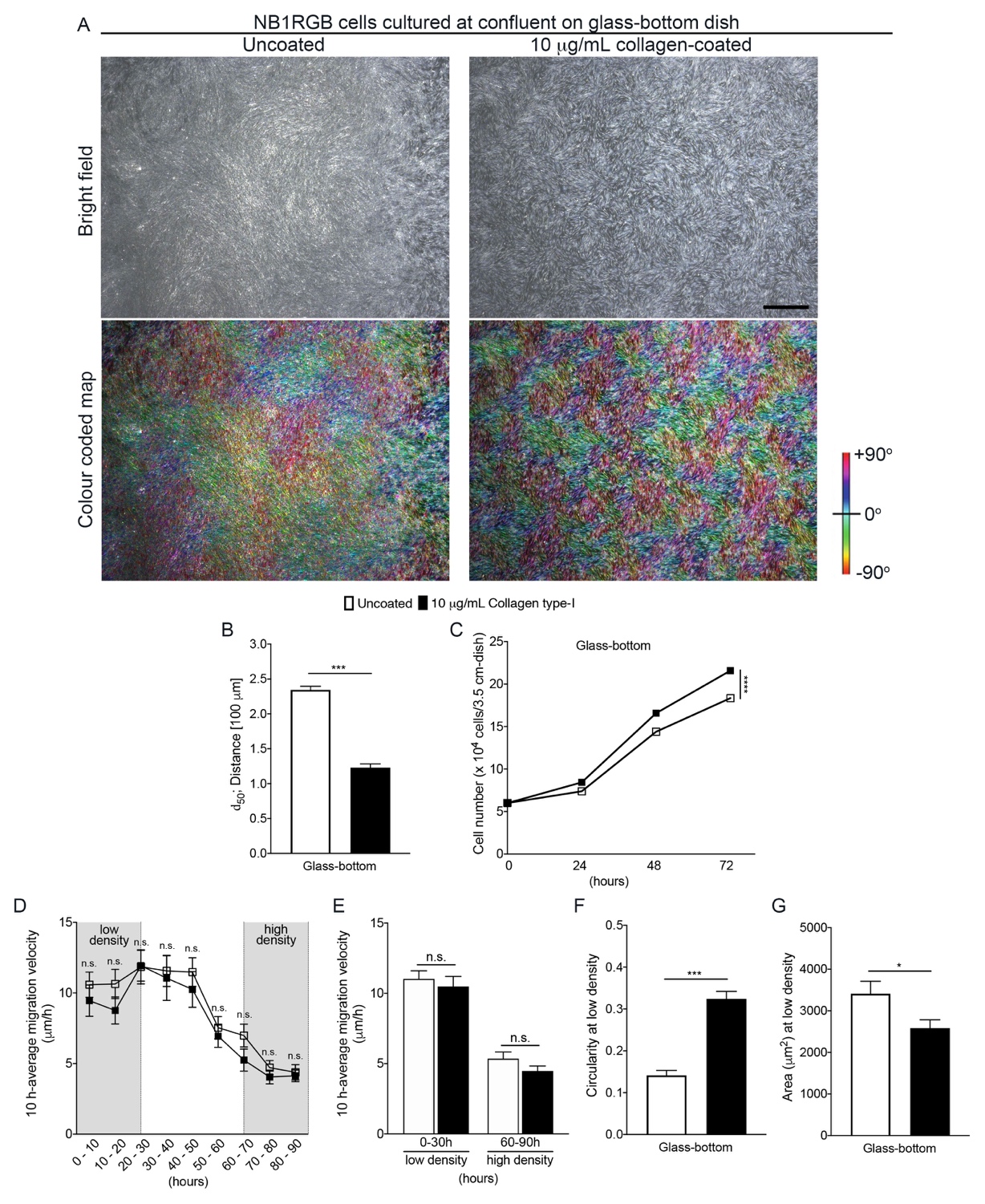


Patterns formed by human-skin NB1RGB fibroblast cells cultured on glass-bottom dishes. (A) Bright-field images and colour coded maps of NB1RGB cultured for 72 h at confluence on uncoated and 10.0 μg/mL collagen type-I-coated glass-bottom dishes. Scale bar: 1 mm. (B) $d_{50}$ value of NB1RGB cells cultured for 72 h. ***: *p* < 0.001, *t*-test. Data represent the mean ± SEM of 4 images from 4 dishes. (C) Number of cells on glass-bottom dishes. ****: *p* < 0.0001, two-way ANOVA. Data represent the mean ± SEM of 4 dishes. (D, E) 10 h-average migration velocity of NB1RGB cultured for 90 h on glass-bottom dishes. two-way ANOVA. Data represent the mean ± SEM of 40 cells at least. (F, G) Circularity and area of NB1RGB morphology at 24 h. *: *p* < 0.05, ***: *p* < 0.001, *t*-test. Data represent the mean ± SEM of 30 cells.

**Supplementary video legends**

**Video 1** Experimentally obtained time courses of cell growth and migration on the uncoated glass-bottom dish.

**Video 2** Experimentally obtained time courses of cell growth and migration on the 10 μg/mL collagen type-I-coated glass-bottom dish.

**Video 3** Numerically obtained time courses of cell growth and migration for *K* = 0.020/h.

**Video 4** Numerically obtained time courses of cell growth and migration for *K* = 0.015/h.

**Video 5** Numerically obtained time courses of alignment process in the absence of spontaneous mobility, reproduction, and excluded volume effect. *K* = 0.020/h.

**Video 6** Numerically obtained time courses of cell growth and migration for *K* = 0.20/h and $\mu=0.10/h$.

**Video 7** Numerically obtained time courses of cell growth and migration *K* = 0.40/h and $\mu=0.10/h$.
